# Development of Dynamic Contrast Sensitivity Chart

**DOI:** 10.22599/bioj.365

**Published:** 2024-07-16

**Authors:** Nikhita Jacob, Vandana Kamath, B. N. Sanjay

**Affiliations:** 1 Assistant Professor, Sankara College of Optometry, Sankara Academy of Vision, Bangalore, India; 2 Optometrist, Sankara College of Optometry, Sankara Academy of Vision, Bangalore, India

**Keywords:** CS function, Dynamic Contrast Sensitivity, Eye movements, Sloan letters

## Abstract

**Background:**

Dynamic visual acuity (DVA) is a complex visual function that requires the observer to detect a moving target, to visually acquire it by eye movements, and to resolve critical details contained in it, in a relatively brief time exposure. Dynamic contrast sensitivity (DCS) functions are determined over a range of angular velocities to complement the traditional contrast sensitivity (CS) functions (obtained with stationary targets).

**Methodology:**

A new chart is constructed to assess DCS by chosen 5×5 grid and Sloan letters (D, H, N, U, V, R, Z, S, K, O, C). Letters are constructed at a constant visual acuity of six lines having the contrast varied at each interval of the line. Each line has six letters and each line subtends different contrast (0.20 logCS–1.70 logCS). The chart has a motor of 45 revolutions per minute (rpm) and 30 rpm and measured among the normal population of the age group of 17 to 30.

**Results:**

Results shows that CS declines once the target stimulus is in motion. There was a statistically significant difference (p < 0.05) between the stimulus speeds of 30 rpm and 45 rpm. Dynamic contrast sensitivity values increased for lower target velocity indicating that as speed of the target stimulus increases, CS decreases.

**Conclusion:**

This study concludes that the DCS decreases as the velocity increases. Consequently, incorporating the DCS chart into comprehensive eye examinations provides a holistic understanding of an individual’s visual function.

## 1. Introduction

Contrast is the amount of luminance or darkness an object has in comparison to its background (Kaur and Gurnami, 2024). Contrast sensitivity (CS) is the ability to perceive sharp and clear outlines and identify the minute differences in the shadings and patterns of objects in the visual space (Kaur and Gurnami, 2024). Contrast is the differences in image intensity whereas the differences in size are called the spatial frequency (Bennett *et al., 2019*). Contrast sensitivity is highly dependent on spatial frequency and this relationship is called the CS function (CSF) (Bennett *et al*., 2019). Pelli *et al*. were among the first to assert that the CSF could be adequately described by two points on the function: visual acuity corresponding to the high spatial cut-off and CS for large letters corresponding to the height of the CSF (Bennett *et al*., 2019; Benjamin and Borish, 1998).

Testing CSF is universally accepted as complementary as it reflects the quality of vision and in cases like glaucoma and diabetic retinopathy declines earlier, while visual acuity remains normal (6/6 or better) in some cases at early stages (Bennett *et al*., 2019). It is an important measure of visual function, especially in situations of low light, fog, or glare due to decreased perception in sharpness of the shadings and patterns of an object (Bennett *et al*., 2019). Drivers at night and Pilots are examples of an individuals who require good CS for safety as there is an increased prevalence of fatal crashes at night, poor steering accuracy, higher self-reports of glare from oncoming headlights, and poorer recognition of road signs (Bennett *et al*., 2019).

Although static visual acuity can be helpful in assessing an individual’s capacity to resolve stationary targets, visual stimuli in everyday life are frequently in motion, particularly while driving a car, operating an aircraft, examining moving objects, or engaging in sports or video games. Dynamic visual acuity (DVA) is defined as a complex visual function that requires the observer to detect a moving target, to visually acquire it by eye movements, and to resolve critical details contained in it in a relatively brief time exposure (Quevedo, Aznar-Casanova and Silva, 2018). According to the committee on the vision of the National Research Council in 1985, DVA measurement is an emergent technique as it is a predictive performance of life in dynamic environment. Recent studies also state that DVA is a key test to investigate vestibular function in otolaryngology especially for elite athletes and drivers (Quevedo, Aznar-Casanova, and Silva, 2018; Rouse *et al*., 1998; Wilkins *et al*., 2013). In the world, we have a dynamic environment and our ability to resolve the targets which are moving shows our performance in a variety of real-world tasks such as driving, flying, or sports activities exposure (Quevedo, L, Aznar-Casanova, JA and Silva, JA da, 2018).

Assessing CSF with dynamic, moving-target testing conditions can lead to more powerful measures of visual assessment that are predictive of visual task performance (Pelli, Robson and Wilkins, 1988; Rao, De and Subhan, 2018). Whereas dynamic CSF are determined over a range of angular velocities to complement the traditional CSF. Visual stimuli in real-life situations often are in motion. Hence a test of an individual’s visual functioning under static conditions may be seriously limited in its ability to predict performance under common dynamic conditions that engage both the optic and oculomotor components of the visual system (Burg, 1966; Long and Homolka, 1992).

The understanding of how complex visual functions can be, especially in dynamic situations, emphasizes the need for a specific tool, such as a dynamic contrast sensitivity (DCS) chart (Bennett *et al*., 2019). This requirement stems from the intrinsic complexity of thorough eye exams, where traditional static evaluations would not be able to adequately capture the subtle differences in CS that occur during dynamic visual tasks (Colenbrander, 2003).

As such, the search for a device with DCS becomes imperative in order to improve the depth and accuracy of ocular exams, which in turn yields a more extensive understanding of visual abilities and supports improved diagnostic and assessment procedures in ophthalmology. A study on DCS used a projector to project the targets of gold-standard static CS Pelli-Robson contrast sensitivity chart (PRCS) onto a front surface mirror mounted on a variable-speed turntable which stresses on the lack of a standard DCS for DCF test (Zavod and Long, 2001).

In day-to-day activities, DVA is essential for adaptation to the dynamic and ever-changing environment in order to predict the future location of stimulus that moves, and anticipatory ability (Quevedo, Aznar-Casanova and Silva, 2018). In a dynamic environment, the position, movement, colour and contrast of the stimulus presented in the visual space keeps changing. Thus, it is essential to understand how well our visual system can detect the CS changing in a dynamic environment but unfortunately it is usually neglected. Therefore, this study aims to construct and validate the new DCS chart.

## 2. Methodology

This is a prospective exploratory study conducted in Sankara College of Optometry in Bangalore, India. Prior to commencing the study, ethical approval was obtained from the Institutional ethics board of Sankara College of Optometry, Bangalore, India.

- **Chart design:** The chart for assessing DCS was constructed using the Sloan letters (D, H, N, U, V, R, Z, S, K, O, C) which fits into 5×5 grid making 5 mic of arc at the nodal point of a person’s eye at 1 meter. Each letter was accurately constructed to ensure uniformity across all the letters in every line. The contrast of the letters varied gradually, with each line containing six letters covering a range from 0.20 logCS to 1.70 logCS in 0.3 logCS steps ensuring that the chart covered a wide spectrum of contrast levels.
- **Motor speed and calibration:** The study was connected to a motor that was set in motion to spin. The motor of the chart was constructed to function at two different motor speeds: 45 revolutions per minute (rpm) and 30 rpm. Prior to the initiation of the data collection, the chart’s functionality was optimized and calibrated for a viewing distance of one meter.
- **Study population:** The target population consisted of university students aged between 17 and 30 years from Sankara college of Optometry, Bangalore, India. A pilot study was done with a total of 40 participants recruited into the study based on inclusion criteria. A written consent was obtained from each participant before enrolling them into the study.
- **Participant selection criteria:** To ensure the integrity of the study results, participants were required to have a minimum static best corrected visual acuity of 0.0 LogMAR (equivalent to 6/6 in Snellen visual acuity chart) for distance in each eye as poor visual acuity can have an impact on the CS. Additionally, participants who had not received any prior training for DVA testing were filtered and included into the study. Subjects with any ocular disorders like anterior or posterior segment diseases, neurological disorders, and eye movements disorders like poor saccades and pursuits were excluded post slit lamp examination, fundus examination, and eye movement tests (Extraocular motility, Saccades, and Pursuits). As none of the subjects had any criteria to be excluded, all the 40 subjects were included into the study.
- **Assessment protocol:**
  - **Baseline evaluation:** A comprehensive assessment was carried forward for each participant which comprises detailed ocular, systemic, and medical histories, visual acuity and refraction for distance and near, slit lamp and fundus examination.
  - **Static CS:** Static CS was measured in static mode monocularly and binocularly using the standard tool, Pelli-Robson chart at one meter post baseline evaluation which was followed by dynamic CS test at static mode, 30 rpm and 45 rpm.
  - **Dynamic CS (DCS):** Assessment of DCS was done using the newly constructed chart in static mode first which was followed by dynamic mode (30 rpm and 45 rpm) both monocularly and binocularly at one meter. Participants were instructed to read through the chart from the lowest contrast level (0.20 logCS) to the highest (1.70 logCS). The test was conducted at a speed of 30 rpm and 45 rpm (once at each speed) and in static mode against a limited time of one minute at each speed.
  - **Data collection and analysis:** Data were collected for each participant and analysed for the effectiveness of the newly developed chart in measuring dynamic CS.

## 3. Statistical Analysis

All the data were analysed using Statistical Package for Social Sciences (SPSS) software version 28. Descriptive analysis was done for age and gender distribution. Paired T – test was used to compare between Pelli-Robson CS chart and constructed DCS chart in static mode. One-way ANOVA was used to compare the mean values between Pelli-Robson CS chart, DCS chart (45 rpm), and DCS chart (30 rpm).

## 4. Results

A total of 40 subjects with a mean visual acuity of 0.0 LogMAR were included in this study. The subjects were between the age group of 17 and 30 years with a mean age of 21.07 ± 2.10 years. Out of 40 subjects 27 were females and 13 were males.

**Figure 1:**
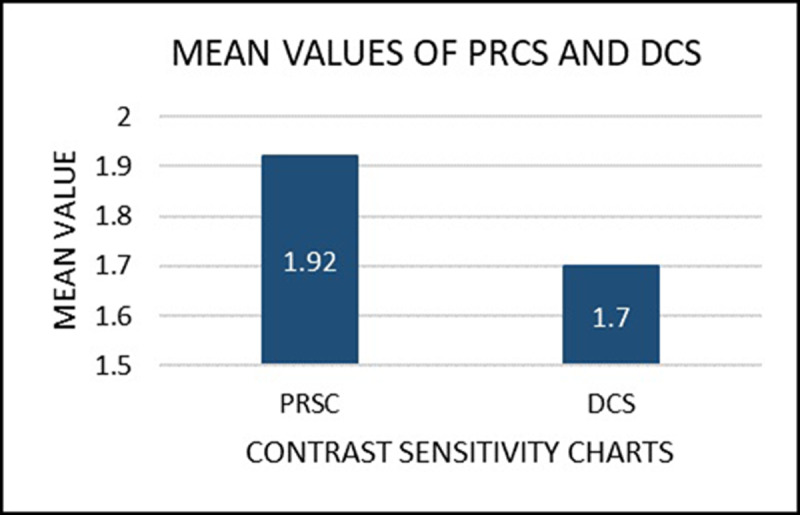
Mean values of PRCS chart and DCS chart in static mode.

On comparing the results obtained from the both the charts PRCS chart and DCS charts in static mode, a prominent difference in the contrast sensitivity value was obtained which was statistically significant with a p-value < 0.05 (Figure 1).

Additionally, a comparison was carried forward among the subjects for the charts PRCS and DCS at different speeds (45 rpm and 30 rpm). The mean values of the CS measurements as recorded by various charts among the subjects is as shown in Table 1. This showed a statistically significant difference with a p-value < 0.001 (Table 2).

**Table 1:** Comparison of PRCS chart to DCS chart.


TEST	MEAN ±SD (OD)	MEAN ±SD (OS)	MEAN ±SD (OU)

**PRCS in static mode (logCS)**	1.92 ± 0.06	1.92 ± 0.06	1.94625 ± 0.06

**DCS chart in static mode (logCS)**	1.7 ± 1.12	1.7 ± 1.12	1.7 ± 1.12

**DCS chart @ 30 rpm (logCS)**	1.7 ± 1.12	1.7 ± 1.12	1.7 ± 1.12

**DCS chart @ 45 rpm (logCS)**	1.3025 ± 0.14	1.3025 ± 0.14	1.3625 ± 0.100


Legends: PRCS: Pelli-Robson contrast sensitivity, DCS: Dynamic contrast sensitivity, rpm: Rotation per minute.

**Table 2:** Comparison of PRCS chart to DCS chart at different rpm.


TEST	EYE	MEAN DIFFERENCE ± SD	P VALUE

**PRCS chart in static mode and DCS chart @ 30 rpm (logCS)**	OD	0.22 ± 1	p < 0.001

	OS	0.22 ± 1	

	OU	0.22 ± 1	

**PRCS chart in static mode and DCS chart @ 45 rpm (logCS)**	OD	0.62 ± 0.08	p < 0.001

	OS	0.62 ± 0.08	

	OU	0.56 ± 0.04	

**DCS chart @ 30 rpm and DCS chart @ 45 rpm (logCS)**	OD	1.398 ± 0.98	p < 0.001

	OS	1.398 ± 0.98	

	OU	1.3375 ± 1	


Legends: PRCS: Pelli-Robson contrast sensitivity, DCS: Dynamic contrast sensitivity, rpm: Rotation per minute.

**Figure 2:**
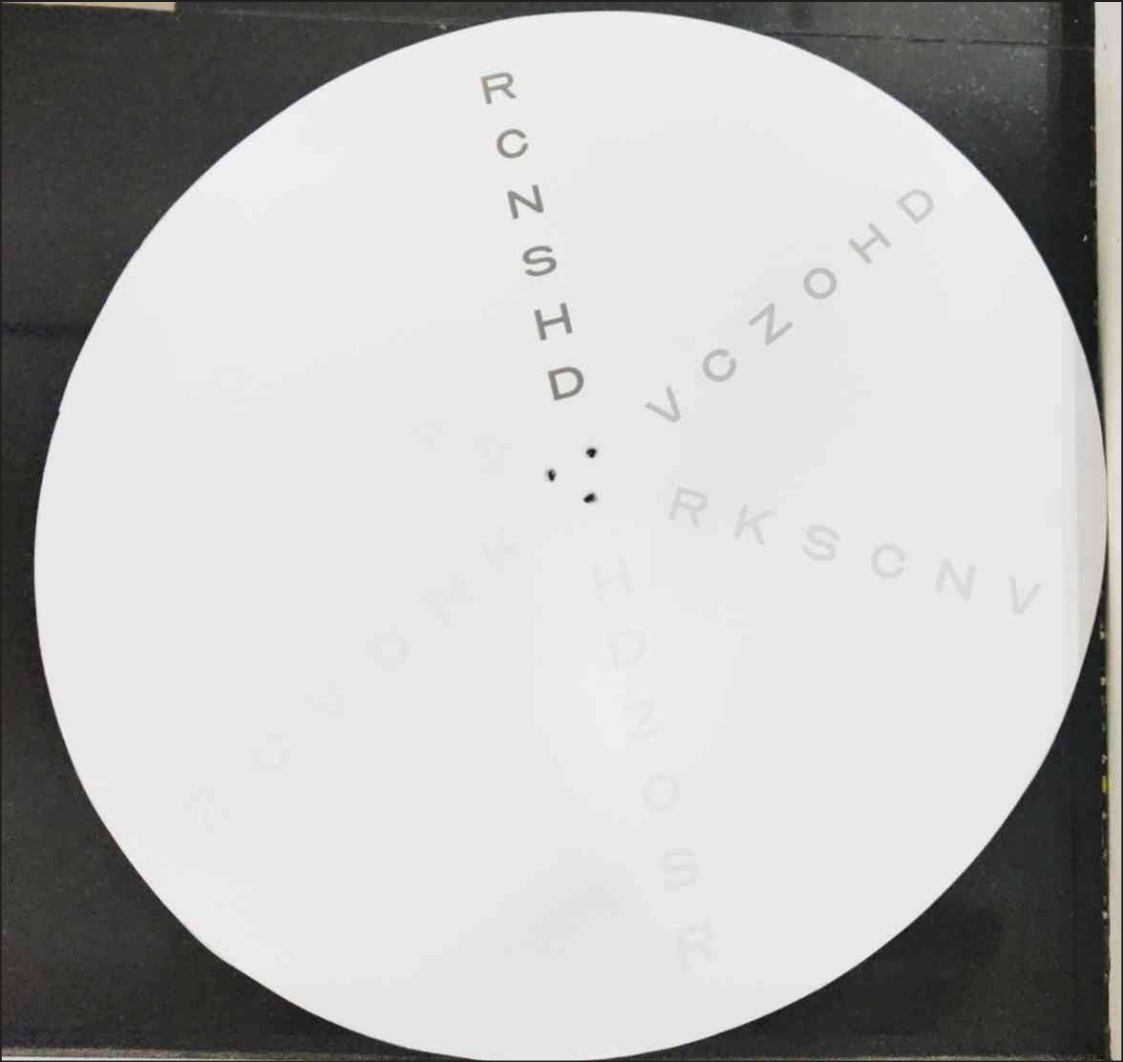
Dynamic contrast sensitivity chart.

This suggests that the newly developed DCS chart (Figure 2), provides dynamic CS measurements at two different speeds (45 rpm and 30 rpm).

## 5. Discussion

This study’s objective was to compare and assess changes in dynamic and static CS using a constructed DCS and a standardized PRCS chart. The study’s goal was also to construct the DCS at two different speeds of 45 rpm and 30 rpm. In this study, there was a significant difference in the contrast sensitivity value with a p-value < 0.05 between the PRCS chart and the constructed dynamic chart when measured in static condition. A possible reason for significant difference is because the maximum the dynamic chart could measure is 1.70 logCS whereas that in PRCS chart is 2.25 logCS.

This study also showed significant decrease in CS scores on increasing the target speed. This result was supported by another study which (Long and Homolka, 1992) employed different target velocities ranging from 30, 60, and 90 deg s-1 and concluded that CS increased significantly for low-frequency targets for 30 and 60 deg s-1 but dropped for high-frequency target velocities. Another study (Long and Zavod, 2002) using letter optotypes found that as target motion is introduced, CS decreases gradually for higher velocities and small targets because CS is inversely proportional to target speed and target size.

Another study (Zimmerman, Lust and Bullimore, 2011) depicts that the dynamic measurements of CS were highly sensitive to conditions like simulated cataracts, especially as target velocity increased. The findings in this study stress on the efficacy of the chart to assess DCS and also demands further investigation and consideration in clinical practice and research settings in order to assess its efficacy on different population like drivers, pilots, sport players.

Therefore, a further assessment and measure of DCS could be beneficial if conducted in the future in a variety of practical applications involving dynamic conditions, such as screening of drivers, athletes, and pilots. Such investigations could also help determine the impacts of age, certain illnesses, or visual system irregularities on DCS. Appropriate management of poor DCS leads to advancements in the quality of life for individuals facing dynamic contrast challenges within their respective fields.

Further studies can be carried out in the future on assessing the DCS among normal people and people with ocular disorders like cataract and glaucoma.

## 6. Conclusion

DCS plays an important role in day-to-day activities like driving, sports. This study concludes that the DCS decreases as the velocity increases. Consequently, incorporating the DCS chart into comprehensive eye examinations provides a holistic understanding of an individual’s visual function.
